# Role of Intraoperative Nerve Monitoring During Parathyroidectomy to Prevent Recurrent Laryngeal Nerve Injury

**DOI:** 10.7759/cureus.880

**Published:** 2016-11-15

**Authors:** Usman Ghani, Salman Assad, Shuja Assad

**Affiliations:** 1 Department of Medicine, Shifa International Hospital, Islamabad, Pakistan; 2 Department of Medicine, Shifa Tameer-e-Millat University, Islamabad, Pakistan; 3 Department of Urology, Nawaz Sharif Medical College, University of Gujrat

**Keywords:** nerve monitoring, parathyroid surgery, recurrent nerve injury, nim, vocal cord paresis

## Abstract

Injury to the recurrent laryngeal nerve (RLN) is a well known, though less frequent, complication of parathyroid surgery. In recent years, the use of intraoperative nerve monitoring (IONM) has gained popularity amongst surgeons when operating on thyroid gland; however, its utilization in parathyroid surgery is not established. This trend continues to rise, despite multiple studies documenting no statistically significant difference that IONM decreases the incidence of RLN injury. Most surgeons use this technology as an adjunct to visualization alone for identification of RLN. The purpose of this review is to discuss the possible role of IONM in parathyroid surgery with regards to the accuracy, efficacy, and recent trends in the utilization of this technology. There is insufficient evidence that IONM reduces the risk of RLN injury in parathyroidectomy. Although IONM may decrease the likelihood of nerve injury by helping to identify and map the RLN during thyroidectomy, we did not find studies exclusive to parathyroid surgery to see if its use can be supported for parathyroidectomy. Despite this lack of evidence, we believe that IONM is a promising adjunct to visualization alone in detecting nerve structures during neck dissection, but more clinical trials are warranted to establish its role in preventing nerve injury in parathyroid surgery.

## Introduction and background

Over the past few decades, the use of an intraoperative nerve monitoring (IONM) system has emerged as a breakthrough in preventing injury to the recurrent laryngeal nerve during neck explorations. It has gained widespread acceptance by surgeons, with up to 40% of endocrine surgeons using this tool during thyroid and parathyroid surgery [1]. The results have been somewhat encouraging for its utilization in thyroid surgery, but whether this modality/technology should be used in parathyroidectomy (PTX) is still arguable. Due to the highly detailed anatomy of the neck, PTX requires meticulous dissection of the next structures in order to reach the pathological gland. In doing so, adjacent neurovascular structures are prone to injury.

**Figure 1:**
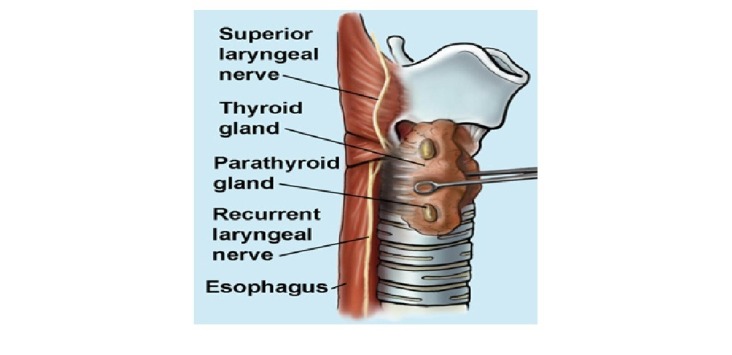
Anatomical Distribution of Recurrent Laryngeal Nerve in the Neck

Recurrent laryngeal nerve (RLN) is vulnerable to injury when neck dissections occur in proximity to the inferior thyroid artery (ITA). The frequency of RLN injury during thyroid and parathyroid surgery has been reported to be up to 3.9% for transient nerve palsy and 3.6% for permanent palsy [2-3]. The anatomic relationship of RLN to the ITA has been described as highly variable; hence, a profound anatomical knowledge is essential during dissection of neck structures [4]. Injury to the RLN is infrequent but detrimental, leading to vocal cord paralysis to temporary or transient loss of voice, swallowing problems, and risk of aspirations. The RLN serves as a landmark to identify pathologic parathyroid glands to be removed as the superior parathyroid glands lie behind the path of the recurrent nerve, whereas the inferior parathyroid glands are located in front of the recurrent nerve path. This anatomical position of the glands in relation to the recurrent laryngeal nerve is of clinical importance for the surgeon to identify the abnormal parathyroid glands. Injuries to the RLN are commonly due to traction, ligation, compression, suture entrapment, and thermal device (Figure 1); however, the rates of nerve injury during PTX are not well documented in the literature [5].

The use of IONM for prevention of RLN injury has remained a controversial subject so far. Multiple studies have compared the outcomes of use of IONM devices to direct visualization of nerve with regards to RLN injury. Most of these studies, to date, involve a patient population with thyroid and parathyroid surgery, and results have been generalized to both procedures as discussed in this review. Studies exclusive to the use of IONM during PTX are not published. We have tried to highlight whether IONM during thyroid surgery can be extrapolated to parathyroid surgery as well and also evaluate the efficacy of nerve monitoring during parathyroidectomy.

## Review

### How does nerve monitoring work?

**Figure 2:**
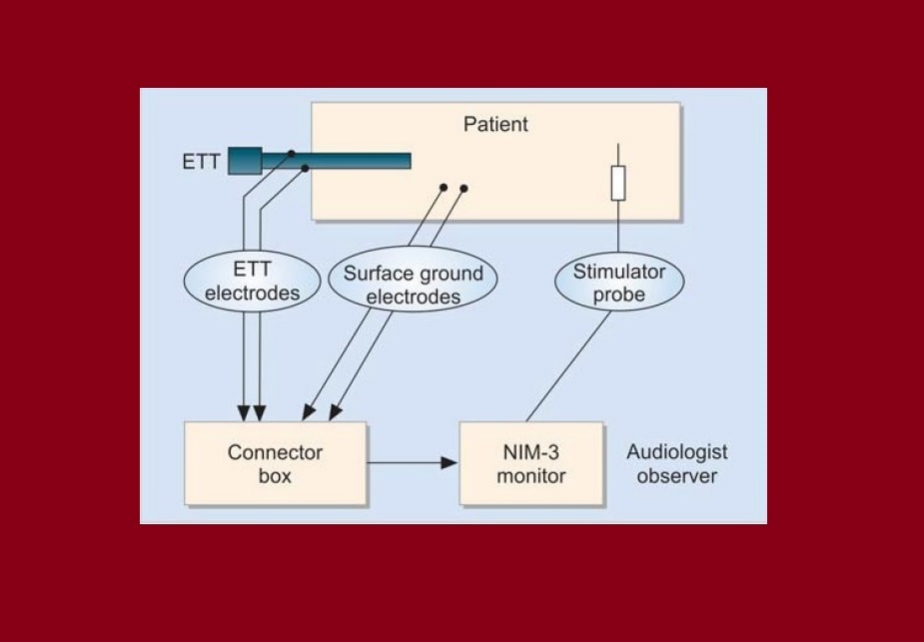
IONM Patient and Equipment Setup Reproduced and modified with permission from Randolph GW: Surgical anatomy of recurrent laryngeal nerve. (*Courtesy* Randolph GW (Ed). In: Surgery Thyroid and Parathyroid Glands. 2003, Saunders, Philadelphia, PA). ETT: endotracheal tube; IONM: intraoperative nerve monitoring; NIM: Nerve Integrity Monitor

IONM of the RLN can be done using various modalities. This includes 1) direct laryngoscope using nerve stimulators, 2) the use of an EMG to monitor the activity of the arytenoid muscles, and 3) endotracheal tube integrated surface probes to record nerve function. The Nerve Integrity Monitor (NIM-Response 3.0 System) (Medtronic Xomed, Jacksonville, Florida) has emerged as a popular device for IONM during thyroid and parathyroid surgery. This nerve monitoring device converts laryngeal muscle action potential into electromyography signals when the RLN nerve is stimulated. The system uses an endotracheal tube with integrated surface electrodes that are placed in contact with the true vocal cords to monitor EMG activity (Figure 2).

**Figure 3:**
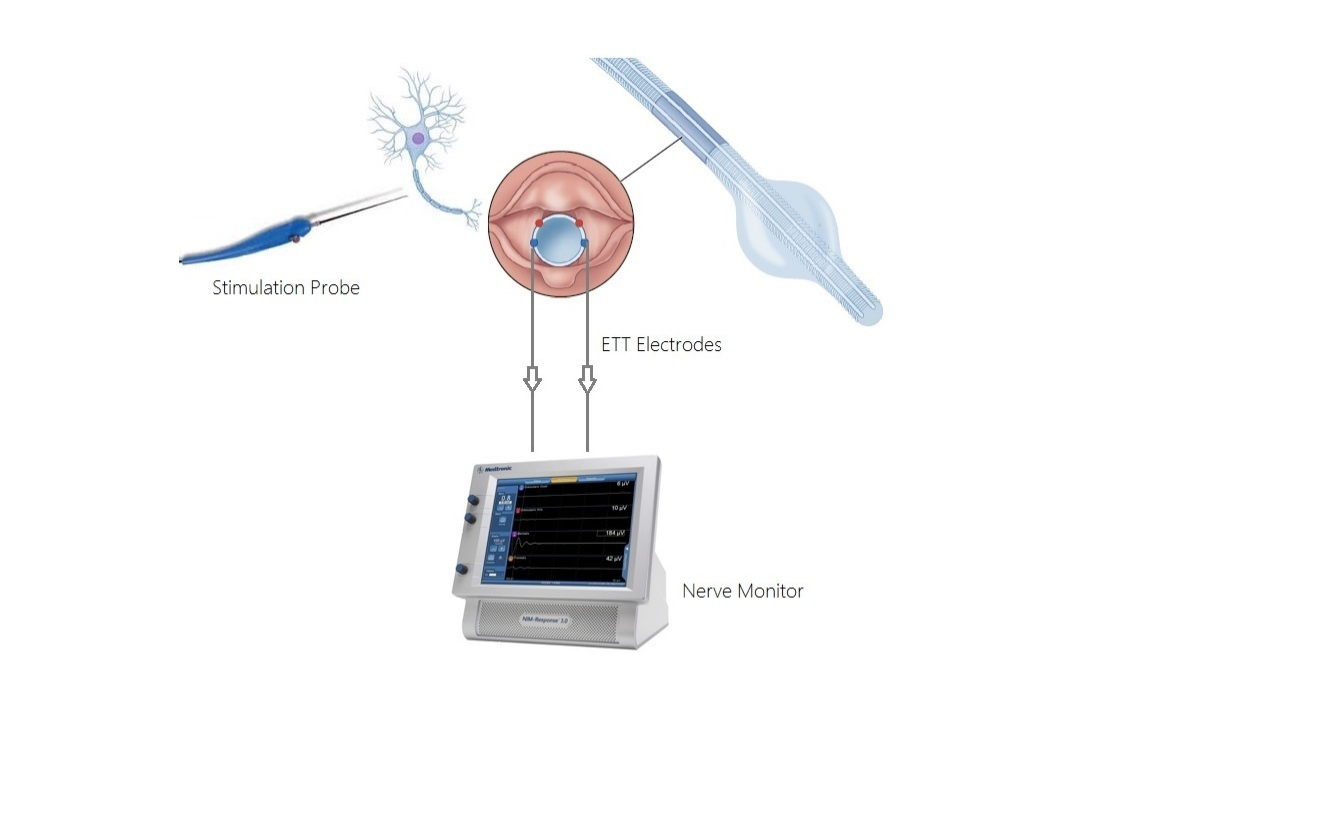
The Medtronic XoMed Tube Nerve Integrity Monitor (NIM) ETT: endotracheal tube

It is important to assess the correct positioning of the electrodes as incorrect placement is a frequent cause of a loss of signal and IONM dysfunction. This is usually done by either direct laryngoscopy or by respiratory EMG waveforms. The ground electrodes wires and the endotracheal surface electrodes are connected to the NIM 3.0 monitor (Figure 3).

A hand-held stimulation probe (monopolar or bipolar) is used to deliver current to the suspected nerve and is connected to the NIM monitor via a connector box. To ensure a functionally intact system, initial vagal stimulation is done by placing the APS (automatic periodic stimulation) probe over the vagus nerve in the carotid sheath. The vagus nerve can be distinguished from the RLN by the latency measurement during EMG responses. An initial stimulation current of 1-2 mA is used to map the nerve, which can be then reduced thereafter. When a functionally intact RLN is stimulated by the probe, an audiovisual warning shows up on the NIM monitor to warn the surgeon with a ‘beep’ and a signal alert is displayed on the monitor. An abnormal EMG response is displayed as a reduction in amplitude and an increase in the latency of waveform on the monitor. This aids in careful dissection, identification, and mapping of the nerve and its branches [5]. In addition to the NIM, laryngeal surface electrodes have been effectively and safely used for RLN monitoring in patients undergoing thyroid and parathyroid surgery [6]. However, in the modern era, less invasive endotracheal tubes have been a substitute for these techniques. 

### What is known so far?

How Accurate Is IONM In Predicting Nerve Injury?

RLN monitoring is frequently used during the thyroid and parathyroid surgery. To justify the use of IONM, the device must be highly sensitive to alert the surgeon during dissection of the neck. A prospective study of 100 patients undergoing thyroidectomy and parathyroidectomy alone or in conjunction with lymph node dissection concluded that IONM was useful in preventing injury to the RLN nerve only if a positive response was elicited on the EMG monitor. In that study, the nerve stimulator aided in the careful dissection of the RLN in 9.2% cases [7]. A retrospective review of the use of IONM in 909 nerves at risk (NAR) reported a sensitivity of 98.4% in predicting normal RLN function when a positive stimulation was observed [8]. In one study including 55 patients undergoing thyroid and parathyroid surgery, the sensitivity of detecting RLN injury by assessing vocal fold mobility was calculated as 75% [9]. Several studies were identified where RLN injury rates were documented to establish the sensitivity and specificity of IONM (Table 1).

**Table 1:** Recurrent Laryngeal Nerve Injury in Various Studies Involving IONM in Parathyroid and Thyroid Surgery (Nerve at Risk Reported for Combined and/or Individual Procedures) *Nerve injured for parathyroid surgery only **IONM; intraoperative nerve monitoring

Year Published	Author	RLN Nerve at Risk	Nerves Injured	% of Nerve Injury vs (% Permanent Nerve Injury)
May 2001	Brennan J [10]	96	1	1% (0%)
Jan 2008	Snyder K [7]	666	25	3.75%
June 2013*	Perie S [11]	100	0*	0 %
October 2013	Eid I [8]	909	-	3.1% (1.2%)
October 2013*	Snyder K [12]	3435	109	3.06% (0.12%)
Feb 2014*	Genther [13]	*90 of 997	22	2.2%

An EMG electrode response is a common modality used during IONM and provides the neurophysiological function of RLN. These electrodes are reliable for monitoring postoperative RLN function. A retrospective observational study assessed the correlation of EMG evoked potentials with postoperative vocal fold function and reported an accuracy of 99.1% when a cutoff EMG amplitude of 200 microV was set [13]. This study is the first to establish the prognostic importance of IONM with a specific EMG amplitude cutoff. Given its high negative predictive value (99.9%), it provides real-time information of RLN function and is a reliable predictor of vocal fold paralysis following thyroid and parathyroid surgery.

What Are The Cost Implications?

Despite its popularity, several centers with low volume endocrine surgery have not adopted the practice of use of IONM. This is possibly due to the fact that IONM remains a debatable subject in terms of the prevention of RLN injury. Various studies have failed to report a statistically significant difference that IONM reduced the likelihood of RLN injury [14]. In addition to this, the economic impact of the technology is another factor that may have contributed to this trend in certain clinical settings. Data about its cost effectiveness in parathyroid exploration, in particular, is unreported, but IONM is associated with increased equipment setup time and increased the cost of operation by 5-7% [15]. Dionigi, et al. argued that IONM decreased the rates of RLN palsy and did not significantly increase the cost of the surgery [15]. Whether the absence of IONM during endocrine surgery in a low volume clinical setup allows a complication-free procedure is again questionable. Richmond, et al. [16] described a single surgeon’s experience of perioperative complication rates in a series of patients undergoing thyroid (n = 131) and parathyroid surgery (n = 19) at a rural hospital without an IONM facility. The overall incidence of RLN injury was reported to be 1.33%. Other parameters, such as identification of parathyroid adenoma and rates of postoperative hypoparathyroidism, were comparable to those reported with the use of specialized technology. Thus, a reasonable opinion stands that a surgeon's experience and skill can possibly bypass the need for IONM and intraoperative technology to prevent complications during thyroid and parathyroid procedures.

If IONM does identify the RLN and reduces the risk of nerve injury, then one might consider the benefits and outweigh the costs of the use of this novel technology, which has at least a 5% increase in the total hospital cost as compared to procedures done conventionally. It could be an option to look at different nerve monitors to reduce the cost of the procedure, such as Neurosign® 1040-4 Channel EMG (Magstim Company Inc., Morrisville, NC), which by far reduces the overall costs when compared to NIM, with comparable accuracy for nerve identification and no difference in complication rates for nerve injury [17].

Is It Really Needed To Prevent RLN Injury?

IONM is considered an adjunctive tool to identify the RLN correctly, thereby allowing additional protection of the RLN during thyroid and parathyroid surgery. It is, by no means, an alternative for careful dissection and visualization of the RLN. Multiple studies have established the role of IONM as a supportive tool to reassure surgeons of RLN integrity. A retrospective review by Yarbrough, et al. emphasized that nerve exposure and direct visualization remain the gold standard techniques in preventing RLN injury during neck surgery [18]. They determined the use of IONM was safe but did not decrease the risk of injury when compared with patients who underwent the procedure without IONM. A systematic review by Dralle, et al. did not find a significant difference in rates of RLN paralysis after thyroid surgery with the use of IONM as compared to those reported for visualization only [14]. IONM did decrease the rates of RLN injury compared to visual identification but were felt to be statistically insignificant. Calò, et al. [19] supported this with his retrospective study that IONM did not decrease the incidence of RLN injury as compared to visualization during dissection but rather provided reassurance to the surgeon of a functionally intact nerve. This raises the question if IONM should be utilized in all cases of neck dissection. A five-year prospective study of 70 thyroid/parathyroid surgery patients (96 recurrent laryngeal nerves at risk) evaluated the efficacy of IONM. The documented rate of temporary paralysis of RLN was 1% and permanent paralysis was 0% in terms of nerves at risk [10].

A single institutional review of 1,936 patients by Snyder, et al. concluded that routine use of IONM for thyroid and parathyroid surgery resulted in a decline in rates of injury to the RLN, in addition to assisting the surgeon in identifying nerves with aberrant anatomy and during difficult dissections for about 10% of nerves [12]. A prospective study by Julien, et al. of patients with thyroidectomy (n = 137) and parathyroidectomy (n = 4) aimed to assess muscle response after RLN stimulation using surface electrodes [20]. The study supported the use of nerve monitoring electrodes to identify the RLN and predict the vocal cord function postoperatively. Therefore, the authors of this review also support the positive outcomes of utilization of a nerve monitor to test nerve integrity. The hand-held stimulation probes used in the NIM 3.0 device allow it to be used for cases where the surgical field is narrow and limited. Kandil, et al. studied the feasibility of using IONM in minimally invasive video-assisted thyroidectomy and parathyroidectomy (MIVAT/P) with emphasis primarily given to the identification of RLN [21]. Marti, et al. published promising results of the use of IONM during minimally invasive thyroid and parathyroid surgery and felt it assisted in a safe identification of the RLN during every step of the surgery [22].

The routine use of IONM in endocrine surgery is increasing. The latest survey comprising of 56 American Association of Endocrine Surgery (AAES) accredited head and neck surgeons revealed that almost 95% of them use IONM in their practice. The common reasons accounted for this widespread use was the surgeons' confidence and improved safety. More than half of the surgeons are likely to modify the extent of surgery on the basis of IONM findings [22]. Another survey by Ho, et al. of 206 surgeons indicated that nerve monitoring was popular amongst head and neck surgeons (HNS) as compared to general surgeons [23]. The HNS performing thyroid and parathyroid surgery used IONM more frequently than general surgeons (80.6% vs 48%), mainly for continuous monitoring during thyroid/parathyroid resection and for medicolegal purposes, whereas general surgeons are more likely to use IONM locating the RLN. However, one survey published by Henry, et al. showed a response predominantly from surgeons who did not routinely use IONM during dissection of RLN during thyroid/parathyroid surgery [24]. Most of these findings were attributed to the level of surgeons' training and the hospital setting where the procedures were performed. A recent online survey of 170 surgeons from five continents assessed the variation in the pattern of use of IONM. This showed a trend that nerve monitoring was utilized more frequently by either younger and less experienced surgeons or those with high volume practice [25].

The use of IONM increases the confidence of the surgeon, in particular, during the early part of their training. This is important as part of tackling complication redo cases, where scarring and adhesions may hinder the visualization of nerve and make it prone to injury with unnecessary manipulation. In this dilemma, the use of IONM has turned out to be a useful technique in minimizing RLN injury, and its use is advocated by some experts in particular cases [26]. Alesina, et al. showed that operative times were longer in patients where intraoperative neuromonitoring (NM) was used and use of adjunct modalities, such as frozen section, was required more frequently in the NM group [27]. The study also supported the experience of the surgeon in preventing complications related to injury to the RLN.

How Does IONM Affect Operative Time?

As earlier mentioned, IONM setup times may increase the equipment setup time. The question of whether it has an impact on the operative time is still being studied. One would argue that the use of specialized technology, such as IONM, should decrease the operative time when compared to the surgeon’s visual capacity to identify RLN. This can potentially decrease the amount of stress of the surgeon. In a study by Sari, et al., all patients undergoing thyroidectomy with the use of IONM had a significantly decreased operative time as compared to those of patients in which IONM was not utilized [28]. However, one retrospective analysis showed increased operative time with the use of IONM, possibly owing to the inexperience of the surgeon, but clearly allowing such surgeons to perform as safe a procedure as they would perform when under the supervision of an experienced surgeon [27]. Also, with revision thyroidectomy, NIM has shown a statistically significant increase in operative times [29]. Périé, et al documented that risk of bilateral RLN paralysis could be minimized by RLN monitoring in bilateral RLN dissection [11]. With the variations in the trend of operative times reported with the use of IONM, it would be reasonable to extend this debate to the parathyroid surgery as well, where established studies are still warranted.

Limitations of IONM

The limitations for the use of IONM during PTX are likely due to the high costs of the nerve monitoring equipment and possible false positive or negative responses. IONM could be considered for parathyroid surgery in which identification of the nerve is difficult, such as redo surgery, radical surgery for parathyroid malignancy involving lymph node dissection, or in cases of anatomical variants of RLN. The extensive dissection involved in such cases may require modification of surgical strategy and approach. The reliability of IONM in facilitating and confirming the identification of RLN, as well as assessing its functional integrity, is based upon its frequent use. Within high volume centers for endocrine surgery, it is reasonable to predict that its utilization will decrease the incidence of recurrent nerve injury in parathyroidectomy. Even though it may be feasible to perform parathyroidectomy without the use of IONM, it is a matter of concern whether the risks involved in medicolegal liabilities outweigh the benefits using the technology for the individual surgeon. However, no medicolegal requirements have been reported yet, so IONM does not mandate an informed consent by law or medical ethics.

## Conclusions

There is no consensus amongst experts whether IONM should be adapted in parathyroid surgery, and its absolute role in utilization in PTX has yet to be established. Visual identification is still regarded as a gold standard in reducing the rate of recurrent laryngeal nerve injury. The use of IONM is a useful adjunct to standard visualization for detection and mapping of the nerve structure. It enables high sensitivity and early warning of nerve damage through real-time feedback. However, further studies are warranted to clearly document the rates of RLN with the use of IONM during parathyroid surgery.
